# Bacterial microbiota from the gut of *Rhodnius ecuadoriensis*, a vector of Chagas disease in Ecuador's Central Coast and Southern Andes

**DOI:** 10.3389/fmicb.2024.1464720

**Published:** 2024-09-23

**Authors:** Juan F. Villacís, Andrea López-Rosero, Juan José Bustillos, Matías Cadena, César A. Yumiseva, Mario J. Grijalva, Anita G. Villacís

**Affiliations:** ^1^Centro de Investigación para la Salud en América Latina, Facultad de Ciencias Exactas y Naturales, Pontificia Universidad Católica del Ecuador, Quito, Ecuador; ^2^Department of Biomedical Sciences, Heritage College of Osteopathic Medicine, Infectious and Tropical Disease Institute, Ohio University, Athens, OH, United States

**Keywords:** Chagas disease, *Rhodnius ecuadoriensis*, gut microbiota, *Trypanosoma cruzi*, habitats

## Abstract

**Introduction:**

Chagas disease is a neglected tropical disease caused by the parasite *Trypanosoma cruzi* that is transmitted mainly by the feces of infected Triatomines. In Ecuador the main vector is *Rhodnius ecuadoriensis* which is distributed in several provinces of the country. More than 40% of these insects in the wild have *T. cruzi* as part of their intestinal microbiota. For this reason, the objective of this research was to characterize the intestinal bacterial microbiota of *R. ecuadoriensis*.

**Methods:**

The methodology used was based on the DNA extraction of the intestinal contents from the wild collected insects (adults and nymphs V), as well as the insects maintained at the insectary of the CISeAL. Finally, the samples were analyzed by metagenomics extensions based on the different selected criteria.

**Results:**

The intestinal microbiota of *R. ecuadoriensis* presented a marked divergence between laboratory-raised and wild collected insects. This difference was observed in all stages and was similar between insects from Loja and Manabí. A large loss of microbial symbionts was observed in laboratory-raised insects.

**Discussion:**

This study is a crucial first step in investigating microbiota interactions and advancing new methodologies.

## 1 Introduction

Chagas disease, also known as American trypanosomiasis, is a zoonotic disease caused by *Trypanosoma cruzi* (Chagas, 1909; Kinetoplastida: Trypanosomatidae) infection. In 2005, it was declared a neglected tropical disease (World Health Organization, 2023) and is endemic in 21 Latin American countries. In Ecuador, as in many other countries where the disease is endemic, it is transmitted by strict hematophagous insects of the Triatominae subfamily through feces or urine contaminated with the parasite *T. cruzi* (Schaub, 2021).

According to the Pan American Health Organization (PAHO) (World Health Organization, 2023), ~70 million people are prone to Chagas disease, with more than 6 million already infected, and 30,000 new cases are reported every year. In Ecuador, a seroprevalence of 0.65% was estimated in the Southern Andes, 1.75% in the Amazon region, and 1.99% on the Central Coast (Dumonteil et al., 2016).

In Latin America, more than 150 species of triatomines have been documented (Justi and Galvão, 2017), while in Ecuador, 16 different species of these insects have been found. Among them, 13 have epidemiological relevance (Chaboli Alevi et al., 2021; Anabel Padilla et al., 2019), with *Rhodnius ecuadoriensis* (Lent and León, 1958) (Hemiptera: Triatominae) being the main vector of the disease (Villacís et al., 2020; Abad-Franch et al., 2001). This species is widely distributed in provinces such as Santo Domingo de los Tsáchilas, El Oro, Guayas, Los Ríos, and Manabí on the coast, in the temperate valleys of Loja, in the Andean Sierra, and in northern Peru (Grijalva et al., 2010; Abad-Franch et al., 2001; Aguilar et al., 1999). Originally, this species of insect was found in wild areas (sylvatic). However, human activities have unintentionally caused environmental changes that allow these bugs to thrive closer to peridomestic and domestic habitats (Grijalva et al., 2005).

The main problem is that triatomines are obligate hematophagous in all their developmental stages, meaning that they need to feed on vertebrate blood to complete their life cycle. Triatomines feed on various blood sources, including *T. cruzi*-infected mammals, which serve as reservoirs for the disease and as a form of vector propagation to humans (Cantillo-Barraza et al., 2020).

*Rhodnius ecuadoriensis* is widely distributed in various environments, with its blood source being domestic animals in the coastal provinces and the highlands of southern Ecuador up to northern Peru. Combined with its synanthropic strength, this makes it one of the main vectors of *T. cruzi* in Ecuador (Grijalva and Villacis, 2009). Their infection rates with *T. cruzi* exceed 40% (Grijalva et al., 2017, 2015), making this species a priority for entomological surveillance.

When *R. ecuadoriensis* feeds on an infected host with *T. cruzi*, it ingests trypomastigotes, which then transform into epimastigotes. These multiply and strictly colonize the insect's midgut, eventually ending up as metacyclic trypomastigotes in the hindgut and spreading through the insect's feces, converting *T. cruzi* into part of the insect's gut microbiome (Rodríguez-Ruano et al., 2018; Soares et al., 2015; Csete et al., 1985).

The set of these microorganisms that develop and reside in symbiosis with a host is considered the microbiome. In the case of the intestinal microbiome, it consists of various members of various kingdoms such as bacteria, fungi, viruses, archaea, and protozoa (Gurung et al., 2019). These microbial communities are highly dynamic and evolve throughout the life of the host. In insects, a close evolutionary relationship between the microbiome and insect is known, although the real extent of the associations is not clearly understood (Gupta and Nair, 2020). Current evidence reveals that these microorganisms act as (i) behavior modulators, (ii) protectors against potential pathogens, (iii) supporters of nutrition, and (iv) facilitators of essential compounds, among other functions (Gupta and Nair, 2020; Dillon and Dillon, 2003).

The microbiota can be classified by its location as “endosymbiont” for microorganisms found inside the insect body or “ecto-symbiont” for microorganisms found on the outside of the insect (Gupta and Nair, 2020). Similarly, microorganisms that may or may not live in association with the insect can be subclassified as: (i) “facultative” or “obligate” for those that strictly need to associate with the insect to survive; (ii) “commensal” for those that benefit from the insect without causing it any harm; and (iii) “parasites” for those that benefit from their association with the insect at the insect's expense (Gupta and Nair, 2020).

The digestive system of *R. ecuadoriensis* serves as an ecological niche for many endo-symbiontic microorganisms (Hypša and Aksoy, 1997; Salcedo-Porras et al., 2020). The composition of the intestinal microbiota varies according to different triatomine species (Arias-Giraldo et al., 2020). In addition, it is influenced by factors such as (i) the type of blood diet, (ii) environmental conditions, and (iii) the presence of competitors, such as *T. cruzi*, which is capable of modulating the insect's immune response in its favor (Castro et al., 2012). In contrast, some bacterial genera are capable of competing with *T. cruzi* for the same ecological niche (Batista et al., 2021) while others even exhibit trypanolytic activities (Castro et al., 2012; Garcia et al., 2010).

Previous studies have been conducted on the intestinal microbiota of other triatomine species (Kieran et al., 2019; da Mota et al., 2012). Other studies have utilized molecular and morphological techniques to investigate the ecology, life cycle, feeding, and defecation patterns, behavior, and population genetics of *R. ecuadoriensis* (Grijalva et al., 2010, 2015; Villacís et al., 2017, 2008). Despite its importance as the main vector of Chagas disease in Ecuador, there has been no previous study characterizing the bacterial microbiota in the gut of *R. ecuadoriensis*. Therefore, understanding the interactions between the intestinal microbiota of *R. ecuadoriensis* and *T. cruzi* is essential for understanding the epidemiology of the disease in Ecuador and proposing possible strategies to interrupt its transmission in endemic areas (Grijalva and Villacis, 2009; Ocaña-Mayorga et al., 2021; Gumiel et al., 2015; Villacís et al., 2010).

With this study, we aim to answer the following questions: (i) What are the predominant bacterial genera found in *R. ecuadoriensis?* (ii) Are there any differences between the microbiota of parasitized triatomines and those that are not parasitized by *T. cruzi?* (iii) Are there any differences between the intestinal microbiota of wild triatomines and those reared in the laboratory? (iv) Does the insect's intestinal microbiota inhibit the development of *T. cruzi?*

## 2 Methodology

### 2.1 Study area

This study was conducted in three rural communities in Ecuador that have a high presence of *R. ecuadoriensis*. The first two communities, Guara and Bellamaría, are located at 1,064–1,450 meters above sea level (MASL) and 1,000–1,384 MASL, respectively. The climate in these communities is characterized by strong interannual variations, particularly influenced by El Nino events. This region also experiences a significant gradient of precipitation, with the eastern slopes of the Cordillera Real receiving over 4,000 mm of rain annually, while the dry valley of Catamayo receives only 300 mm (Rollenbeck et al., 2006). The third community, Caja Fuego (~100 masl), is located in Portoviejo County, Manabí province. Its climate is a mix of dry subtropical and humid tropical (Grijalva et al., 2017).

### 2.2 Triatomine collection

**Figure 1:**
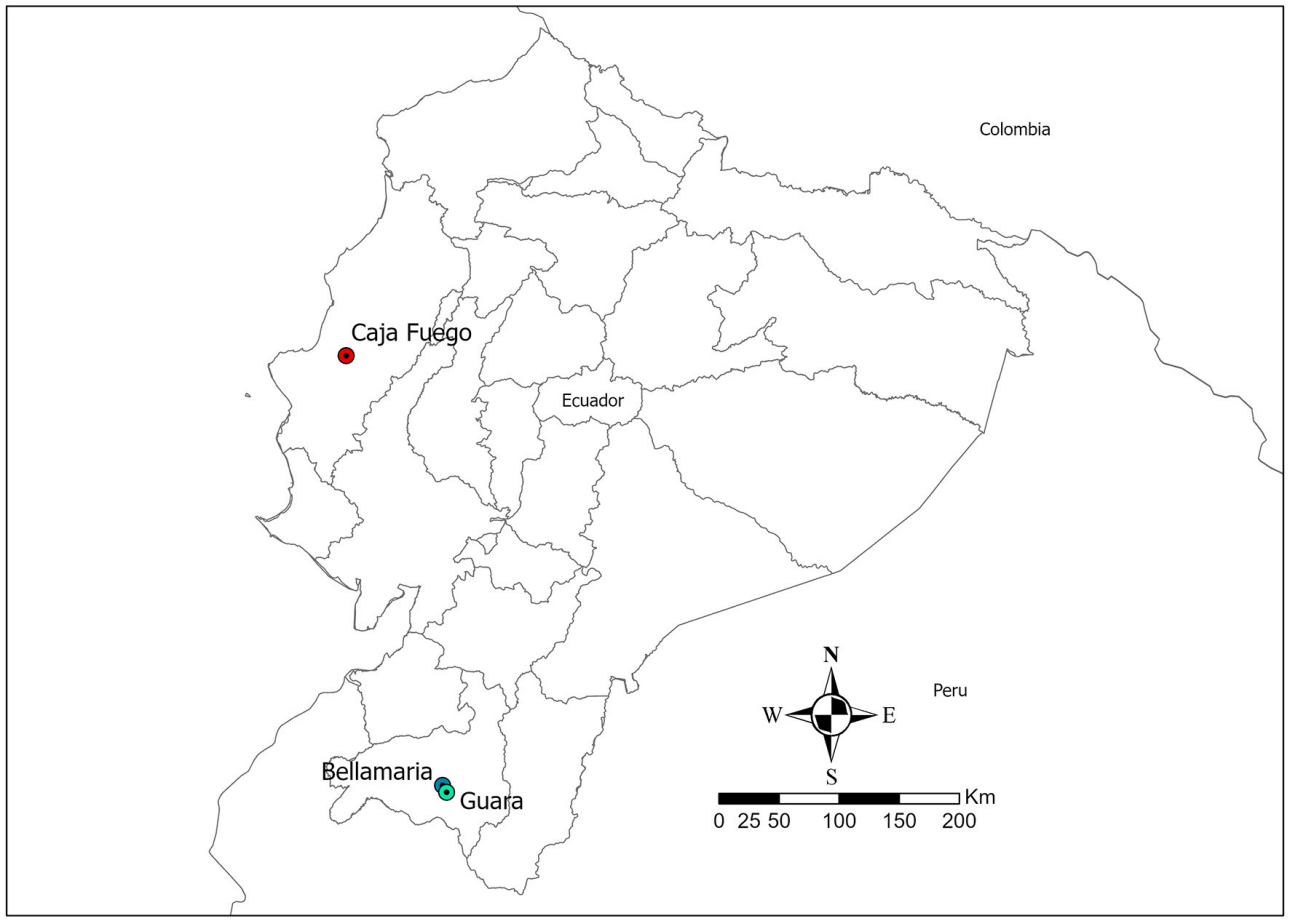
Map of Ecuador showing the location of the communities: Caja Fuego community, Central Coastal Region (Manabí Province), and Bellamaría and Guara communities, Southern Andes (Loja Province).

The collection of triatomines will be conducted in the coastal province of Manabí and the Andean province of Loja in Ecuador, with collection permits 002-17IC-FAU-DNBAPVS/MA, 010-IC-FAU-DNBAPVS/MA, and MAAE-DBI-CM-2021-0185, respectively (Figure 1). Domiciles, peridomiciles, and sylvatic areas will be searched for triatomines using previously described methods (Grijalva et al., 2005; Grijalva and Villacis, 2009; Villacís et al., 2017). Both live and dead insects will be collected and transported in sterile vials with the use of mobilization permit number MAAE-CMARG-2020-0178 to the Center for Research for Health in Latin America (CISeAL). The identification of developmental stages and sex (of adults) of the collected triatomines will be performed using a dichotomous key by Lent and Wygodzinsky (1979).

### 2.3 Laboratory triatomines

The insect colonies were established using specimens collected in previous years (insects from Loja in 2019 and Manabí in 2023) from various communities in Ecuador, including those previously mentioned. Each colony consisted of insects from a single community and sampling point. The foundation of the colonies was established among adults. If we consider that *R. ecuadoriensis* needs at least 6 months to complete its life cycle (Villacís et al., 2008), at least two or more generations occurred in Loja colonies and only one in Manabí, where there may have been contact between the newly hatched nymphs and parents. The insects were maintained under controlled conditions of humidity, temperature, and photoperiod as described by Villacís et al. (2008). They were periodically fed with human blood every 15 days using the Hemotek membrane feeding system (Hemotek Ltd., Blackburn, UK) for 45 min (Gysin et al., 2022; Luis Costa-da-Silva et al., 2014).

### 2.4 Extraction of intestinal contents (ICs) and culture media

Each insect was placed in a sterile tube at −20°C for 20 min and then rinsed with 70% ethanol and sterile distilled water to remove dirt and the accompanying microbiota (Montoya-Porras et al., 2018). Immediately, the ICs were extracted under aseptic conditions in a laminar flow chamber, and a transverse cut was made at the genitalia tip with a sterile scalpel. Hindgut and feces were removed by using a variable-volume micropipette and sterile filter tips. As part of the screening process, the ICs were inoculated in different general and differential culture media, such as blood agar, nutrient agar, SS agar, salted Mannitol agar, and MacConkey agar.

### 2.5 Deoxyribonucleic acid (DNA) extraction and polymerase chain reaction (PCR) amplification

DNA extraction was performed using the ZymoBIOMICS DNA Microprep Kit based on the protocol established by the manufacturer, with the following variations: The intestinal content was recovered directly in the lysis solution, and 10 ul of proteinase K was added, followed by incubation at 55°C for 50 min and centrifugation at ≥10.000 rpm for 1 min. DNA concentration was measured using a Nanodrop 2000c (Thermo Fisher Scientific, Waltham, MA, USA). The bacterial 16S DNA was amplified with the primers F27 and R1492 using the GoTaq Flexi DNA Polymerase Kit and dNTP Mix (Promega). Amplification made by conventional PCR started with 5 min of initial denaturation at 95°C, followed by 35 cycles of 1 min of denaturation at 95°C, 1 min of hybridization at 59°C, and 1 min elongation at 72°C, with a final elongation of 10 min at 72°C (Heuer et al., 1997). Similarly, for the amplification of DNA of *T. cruzi*, TcZ1, and TcZ2 primers were used with 3 min of initial denaturation at 95°C, followed by 40 cycles of 20 s of denaturation at 95°C, 15 s of hybridization at 59°C, and 30 s elongation at 72°C, with a final elongation of 7 min at 72°C (Moo-Millan et al., 2019). Finally, a 1.5% agarose electrophoresis was performed to confirm the presence of DNA.

### 2.6 Pools

For the formation of pools, only live insects selected by the province, community, habitat, and sex were used. This was done to study a larger part of the bacterial microbiota of *R. ecuadoriensis* and obtain an overview of the bacterial intestinal microbiota of each group of triatomines (Schisterman and Vexler, 2008).

### 2.7 Metagenomics amplicon

Amplicon sequencing was performed using extracted DNA that had been previously quantified and grouped into pools. The amplicons were sequenced on the Illumina MiSeq platform (Biosequence S.A.S., Quito, Ecuador) after the library was constructed with primers targeting the hypervariable regions V3–V4 of the 16S ribosomal DNA. The primer pairs used were 341F: (5′-CCTACGGGNGGCWGCAG-3′) and 805R: (5′-GACTACHVGGGTATCTAATCC-3′).

### 2.8 Bioinformatic analysis

The Fastq files from each pool were subjected to a quality and filtering process to guarantee accurate taxonomic classification. For the taxonomic classification, a high-performance algorithm of the Ribosomal Database Project (RDP) classifier described by Wang et al. (2023) was used. The database used is RefSeq RDP 16S v3, based on a set of Fast Alignment Search Tool (FASTA) format files from: https://benjjneb.github.io/dada2/training. These files contain 16S ribosomal ribonucleic acid (rRNA) gene sequences in Divisive Amplicon Denoising Algorithm 2 (DADA2) (Callahan et al., 2016) format for bacteria and archaea (Version 2). After this, the analysis included (i) calculating species richness (using the Margalef index), (ii) determining Shannon α diversity index and β diversity, (iii) calculating Simpson's dominance index, and (iv) calculating Simpson's diversity index for genera and species. Rarefaction curves were also generated using iNEXT Online (version March 2024) (Chao et al., 2016). In addition, the data were analyzed using the Mann–Whitney test in the Statistical Package for the Social Sciences (SPSS) software (version 29.0.2.0).

## 3 Results

The intestinal content of *R. ecuadoriensis* inoculated in non-differential culture media demonstrated the presence of bacteria. We observed bacterial growth after 48 h at 28 ± 3°C only in blood agar. The PCR results, visualized by electrophoresis, showed that 41.66% and 25.57% of the insects collected in Loja and Manabí, respectively, were infected with *T. cruzi* as part of their intestinal microbiota (data not shown).

### 3.1 Bacterial genus composition of wild collected *R. ecuadoriensis*.

In this study, a total of 45 *R. ecuadoriensis* specimens were collected from the wild and divided into six pools according to their stage and location. The predominant bacterial genera found in the intestines were *Corynebacterium* (20%), *Eikenella* (14.09%), *Rhodococcus* (11.65%), *Williamsia* (5.80%), *Enterococcus* (3.95%), *Staphylococcus* (2.53%), and *Yokenella* (2.39%) (Table 1). Genera with an abundance of <1% were grouped as “other.” However, the main species identified in the intestine were *Corynebacterium glycinophilum* (23.66%), *Eikenella corrodens* (20.14%), *Williamsia serinedens* (8.39%), *Enterococcus faecalis* (5.68%), *Corynebacterium terpenotabidum* (4.02%), *Rhodococcus marinenascens* (3.90%), *Staphylococcus xylosus* (2.35%), and *Snodgrassella alvi* (1.34%). These results showed significant statistical differences (*p* = <0.001) between the groups analyzed (Manabí and Loja), as calculated by the Mann–Whitney test.

**Table 1:** Bacterial microbiota's (bacteria genus) composition of nymphs and adults of wild *R. ecuadoriensis*.

**Genus**	**Loja male %**	**Loja female %**	**Loja nymph V %**	**Manabí male %**	**Manabí female %**	**Manabí nymph V %**
*Arsenophonus*	3.34^*^	0.11	0.13	0.17	0.48	0.38
*Corynebacterium*	2.31^*^	31.23^*^	74.52^*^	0.09	0.24	64.67
*Eikenella*	2.00	37.24	0.04	17.50^*^	64.04^*^	0.01
*Rhodococcus*	26.13^*^	0.05	0.04	0.18	0.00	0.03
*Williamsia*	0.41	0.00	0.04	46.77^*^	0.00	3.77^*^
*Yokenella*	0.85	8.05^*^	0.00	10.90^*^	4.04	0.00
*Enterococcus*	0.05	1.48^*^	0.00	10.73^*^	0.11	23.06^*^
*Staphylococcus*	0.21	0.01	21.84^*^	0.00	0.02	0.02
*Morganella*	1.62	3.94^*^	0.02	0.01	0.05	0.05
*Snodgrassella*	0.01	2.13	0.00	0.98^*^	4.93^*^	0.02
*Propionibacterium*	0.51^*^	2.34^*^	0.01	0.02	0.00	0.00
*Phyllobacterium*	2.94^*^	0.33	0.03	0.13	0.33	0.01
*Gordonia*	0.01	0.01	0.01	0.29^*^	0.00	3.62^*^
*Lactococcus*	0.21	0.02	0.00	1.12	0.02	0.00
*Povalibacter*	1.23^*^	0.00	0.00	0.00	0.00	0.00
Unclassified at the genus level	39.39	10.86	2.20	9.36	22.84	1.93
Other	18.78	2.20	1.13	1.73	2.91	2.43

Stage: Males (M), Females (F), Nymph V (NV). ^*^Significant differences.

### 3.2 Comparison of the bacterial microbiota's (genus of bacteria) *R. ecuadoriensis*: bacteria wild collected vs. laboratory-reared

**Figure 2:**
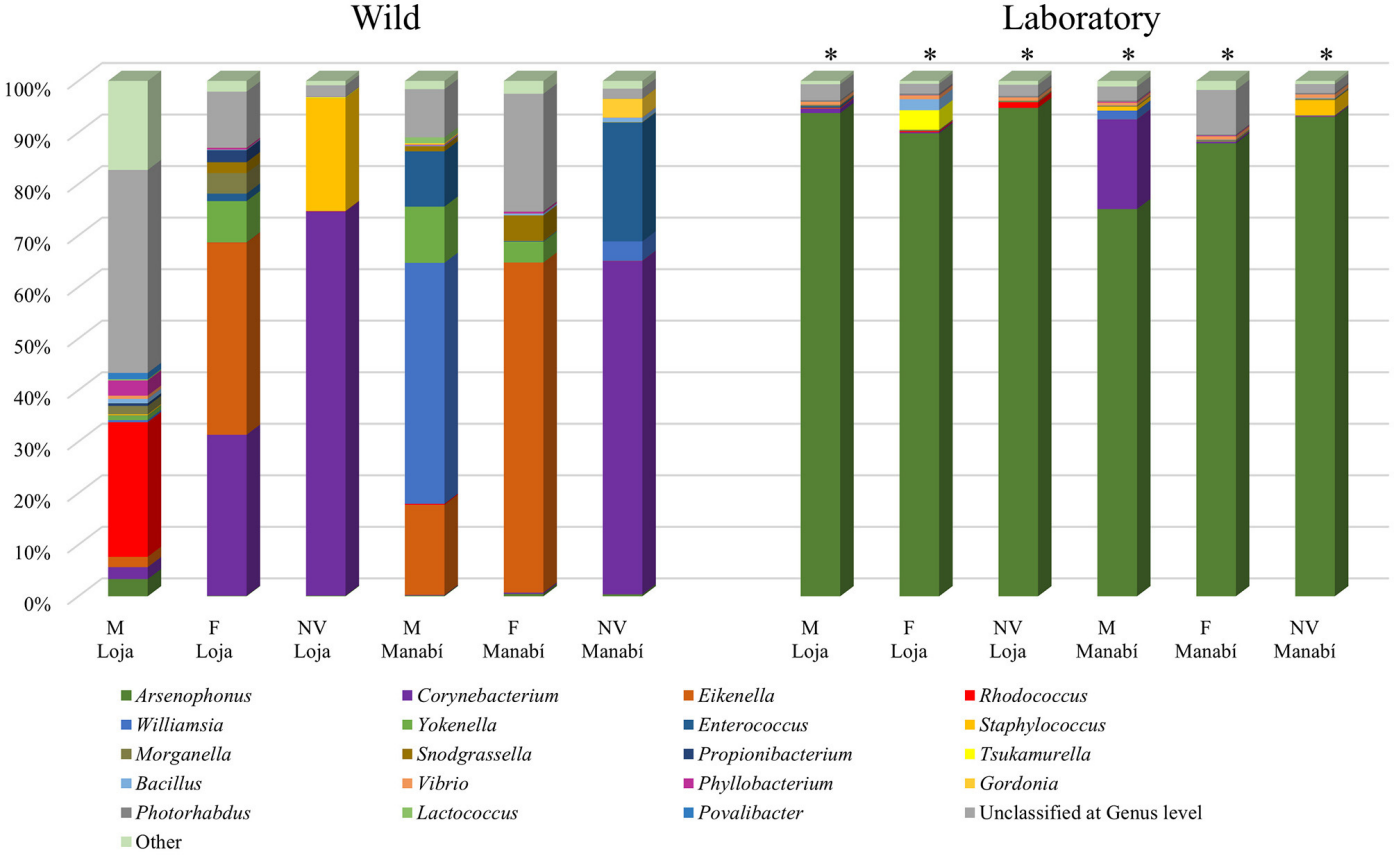
Comparison of the bacterial microbiota (genus of bacteria) of nymphs and adults of wild collected and laboratory-reared *R. ecuadoriensis*. Stages: Nymph V (NV), Females (F), Males (M).

The diversity of bacterial genera in the intestinal microbiota of *R. ecuadoriensis* showed significant differences (*p* ≤ 0.001) between wild collected (6 pools) and laboratory-reared (6 pools) insects. In the wild collected group, the most abundant bacterial genera in wild insects were *Corynebacterium* (20%), *Eikenella* (14.09%), *Rhodococcus* (11.65%), *Williamsia* (5.80%), *Enterococcus* (3.95%), *Staphylococcus* (2.53%), and *Yokenella* (2.39%), totaling 7 genera. The laboratory-reared insects had a different composition, with *Arsenophonus* (89.07%) and *Corynebacterium* (3.14%) being the most abundant genera (2 in total). The “other” category in Figure 2 represents the sum of all genera with <1% abundance.

### 3.3 Diversity indices for genera and species

**Figure 3:**
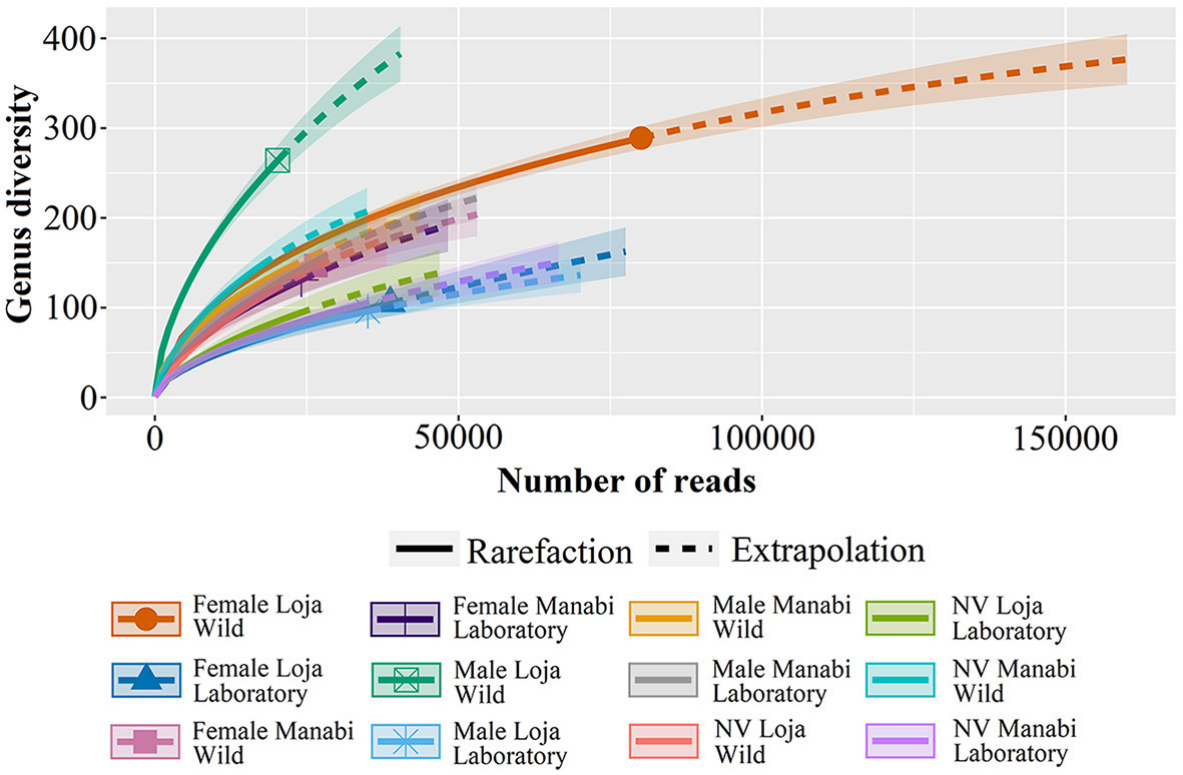
The rarefaction curve shows the relationship between the number of sequenced reads and genera diversity in different groups. The “Female Loja Wild” group exhibits greater gender diversity, while others, such as laboratory samples, reach saturation with fewer readings.

Figure 3 presents a rarefaction curve that explains the relationship between the number of sequenced reads and the diversity of genera in different groups. The results indicate that samples such as “Female Loja Wild” exhibit a higher gender diversity, while others, such as laboratory samples, reach saturation with fewer readings.

Table 2 displays the diversity indices, with the female from Loja Wild showing the highest species richness (Margalef index = 26.128). The Shannon α index for the bacterial genera and species present in wild and laboratory-reared *R. ecuadoriensis* also showed differences. Wild insects have a moderate diversity of genera and species, with an index >1. However, the nymphs V (NV) from Loja showed a low diversity of genera, with an index of <1. Similarly, the female (F) from Manabí has a low diversity of species, with an index of <1. On the other hand, the insects from the laboratory-reared populations in both Loja and Manabí have low or very low diversity, with indices of <1 and 0.5, respectively.

**Table 2:** Diversity indices (Species Richness, Shannon α, Simpson‘s Dominance, and Diversity) for bacterial genera and species found in wild and laboratory-reared *R. ecuadoriensis*.

**Wild insects of Loja**	**Loja wild**						**Loja laboratory**					
	**Genera**			**Species**			**Genera**			**Species**		
	**M**	**F**	**NV**	**M**	**F**	**NV**	**M**	**F**	**NV**	**M**	**F**	**NV**
Species richness (Margalef index)	0.000	0.000	0.000	22.593	26.128	9.739	9.078	9.844	9.143	6.307	6.436	5.764
Shannon alpha (α–diversity)	1.175	1.750	0.757	1.232	1.478	0.977	0.364	0.530	0.318	0.313	0.424	0.268
Beta diversity (β) (compared between Loja and Manabí)	0.632	0.472	0.430	0.219	0.301	0.222	0.376	0.373	0.400	0.822	0.299	0.231
Beta diversity (β) (compared between wild and laboratory)	0.317	0.365	0.284	0.151	0.208	0.141	0.317	0.365	0.284	0.151	0.208	0.141
Simpson's dominance index	0.947	0.423	0.166	0.265	0.395	0.196	0.019	0.039	0.014	0.025	0.053	0.020
Simpson's diversity index	0.053	2.362	0.834	0.735	0.605	0.804	53.527	25.447	73.321	0.975	0.947	0.980
**Wild insects of Manab**í	**Manab**í **wild**						**Manab**í **laboratory**					
	**Genera**			**Species**			**Genera**			**Species**		
	**M**	**F**	**NV**	**M**	**F**	**NV**	**M**	**F**	**NV**	**M**	**F**	**NV**
Species richness (Margalef index)	14.314	14.333	14.946	12.112	11.290	12.080	15.024	12.883	9.701	9.328	8.324	5.859
Shannon alpha (α-diversity)	1.660	1.182	1.152	1.400	0.939	1.606	0.895	0.562	0.394	0.825	0.458	0.357
Beta diversity (β) (compared between Loja and Manabí)	0.632	0.472	0.430	0.219	0.301	0.222	0.376	0.373	0.400	0.822	0.299	0.231
Beta diversity (β) (compared between wild and laboratory)	0.356	0.367	0.386	0.697	0.199	0.199	0.356	0.367	0.386	0.697	0.199	0.199
Simpson's dominance index	0.390	0.237	0.232	0.394	0.234	0.427	0.156	0.060	0.022	0.164	0.071	0.026
Simpson's diversity index	2.565	4.218	4.315	0.606	0.766	0.573	6.426	16.624	44.903	0.836	0.929	0.974

Stage: Nymph V (NV), Females (F), Males (M).

The Simpson's Diversity Index also showed differences between wild and laboratory-reared populations. In wild *R. ecuadoriensis*, the males from Loja exhibited low diversity in genera, with an index lower than 0.3. However, the females from Loja and males and NV from Manabí showed moderate diversity in species, with indices ranging from 0.34 to 0.66. All other populations, both wild and laboratory-reared, showed high diversity, with indices >0.67 (Table 2).

### 3.4 Distribution of bacterial genera in the absence and presence of the parasite, *T. cruzi*

The most abundant intestinal microbiota of *R. ecuadoriensis* infected with *T. cruzi* was composed of the genera *Corynebacterium* (32.85%), *Williamsia* (15.59%), *Eikenella* (7.69%), *Enterococcus* (5.01%), *Yokenella* (3.63%), and *Povalibacter* (1.23%) (Table 3). Contrastingly, insects that did not have *T. cruzi* as part of their microbiota showed the genera *Eikenella* (29.37%), *Rhodococcus* (12.99%), *Corynebacterium* (11.18%), *Yokenella* (5.12%), and *Enterococcus* (3.91%). Other abundant genera included *Morganella* (2.38%), *Snodgrasella* (1.89%), *Phyllobacterium* (1.69%), and *Propionibacterium* (1.43%). However, the Mann–Whitney test did not show significant statistical differences between the groups analyzed (p = 0.229).

**Table 3:** Distribution of bacterial genera in the absence and presence of *Trypanosoma cruzi*.

**Genus**	***T. cruzi* (+) %**	***T. cruzi* (–) %**
*Arsenophonus*	3.37	0.21
*Corynebacterium*	32.85	11.18
*Eikenella*	7.69	29.37
*Enterococcus*	5.01	3.91
*Morganella*	0.83	2.38
Other	14.88	14.88
*Phyllobacterium*	0.05	1.69
*Povalibacter*	1.23	0.00
*Propionibacterium*	0.01	1.43
*Rhodococcus*	0.28	12.99
*Snodgrassella*	0.33	1.89
*Staphylococcus*	0.00	0.12
Unclassified at the genus level	14.24	23.69
*Williamsia*	15.59	0.83
*Yokenella*	3.63	5.12

## 4 Discussion

We present a pioneering study of characterizing the bacterial microbiota from the gut of *R. ecuadoriensis* using pools for amplicon metagenomics. This technique allows for the analysis of a greater number of sequences in less time and at a lower cost compared to a traditional methodology using isolation in culture media, which has limitations, such as a limited number of culturable bacteria due to the specific nutritional requirements and conditions necessary for their growth in the laboratory. In this way, a greater panorama of the intestinal bacterial symbionts of *R. ecuadoriensis* was obtained, providing valuable information on the biology of the insect and its implications for the vectorial transmission of *T. cruzi*.

The different insect groups (stages, habitats, and provinces) showed a bacterial diversity that is not found in other insects. The representative genera of this study were *Arsenophonus, Corynebacterium, Eikenella, Rhodoccoccus, Williamsia, Yokenella, Enterococcus*, and *Staphylococcus*, which represent more than 60% of the bacterial microbiota of *R. ecuadoriensis*. Our results showed a differentiation between bacterial genera in the different stages of wild insects. This confirms that the digestive system of *R. ecuadoriensis* is a dynamic microhabitat, and its microbiota is directly related to the (i) insect stage, (ii) the environmental conditions where they come from and where they develop, and (iii) the presence of certain microbial groups (Guarneri Alessandra and Schaub, 2021), as previously reported for other species mentioned by Muñoz-Benavent et al. (2021).

There is a significant statistical difference in the bacterial composition between wild and laboratory-reared triatomines. Our results indicate that laboratory-reared *R. ecuadoriensis* had a higher predominance of the *Arsenophonus* genus (Loja = 94.11% and Manabí = 85.35%). There are several ways in which the insects can acquire their intestinal microbiota, starting with hatching, contact with environmental microorganisms, hematophagy, coprophagy, and cannibalism (Guarneri Alessandra and Schaub, 2021). Therefore, we suggest that the loss of diversity of insects raised in the laboratory is due to: (i) controlled environmental conditions, (ii) the type of food (blood) and how they are fed in the laboratory, and (iii) lack of contact with different microorganisms (Schaub, 2024). Accordingly, only wild insects can provide accurate information about the intestinal bacterial microbiota of *R. ecuadoriensis*.

Contrary to what was expected, in this study, we did not identify the *Wolbachia* genus in any of the samples analyzed. *Wolbachia* is one of the well-studied bacterial genera and is commonly found in different insects. It is known that *Wolbachia*'s function can modify the behavior of the insect in its favor to promote its transmission (Lewis and Liz, 2015). In contrast, in all groups (wild and laboratory-reared), we noticed the presence of the *Arsenophonus* genus, which has similar mechanisms to *Wolbachia* to favor its transmission (Lewis and Liz, 2015). In this study, we discovered the presence of *Eikenella*, an opportunistic bacterium typically found in the human oral cavity and upper respiratory tract. All necessary biosafety protocols, including the use of masks, gloves, and glasses, were strictly followed during insect handling and DNA extraction processes. Notably, Wertz and Breznak (2007) identified a bacterium that is 94.1% similar to *Eikenella corrodens* in the termite guts, suggesting that this species could begin to adapt to other organisms.

As suggested by Durvasula et al. (2008), *Corynebacterium* is a symbiotic bacterium found in triatomines. It provides pantothenic acid to the nymphs and is necessary for their development and maturation. This is supported by the higher presence of this bacterial genus in wild NV nymphs of *R. ecuadoriensis*. Interestingly, we also found the presence of the genus *Xenorhabdus* in all the samples analyzed. This bacterium is a symbiont of nematodes from the family Steinernematidae (Rhabditida), which are known to be obligate endopathogens of insects (Ruiz Laparra, 2019). In addition, the genus *Lactococcus* was only present in adult insects, both male and female. Conversely, the genus *Nocardia* was only found in NV nymphs; nevertheless, the function of these last two genera of bacteria is unknown.

The species *Rhodococcus rhodnii* was identified as a symbiont of *Rhodnius prolixus*. Its function is to produce cecropin A, which significantly reduces the parasite load of *T. cruzi* in the insect and also provides the insect with vitamin B (Lage et al., 2023; Kollien and Schaub, 2000). In this study, *Rhodococcus* was present in 2.9% of non-infected individuals and only 0.28% of infected individuals. In addition, our results indicated that the species *Serratia marcescens* is present in six groups of samples. This has been reported as a symbiont of hematophagous insects by Vieira et al. (2018), and some strains have been found to intrinsically possess trypanolytic activity (Rodríguez-Ruano et al., 2018), giving light to future research for improved *T. cruzi* transmission control.

Rodríguez-Ruano et al. (2018) describe *Arsenophonus triatominarum* as a bacterial symbiont in other triatomine species, particularly *Triatoma infestans*. In our study, we found the presence of *Arsenophonus nasoniae* in all samples, which raises the question of whether this species is a natural symbiont of *R. ecuadoriensis*. Montoya-Porras et al. (2018) noted that *Williamsia* is one of the most prevalent bacteria in *Rhodnius pallescens*. This bacteria may play a significant role as a symbiont in triatomine species and could be useful in paratransgenic studies. This observation raises the question of whether *Williamsia* is specific to the *R. pallescens* complex, which includes *R. colombiensis, R. ecuadoriensis*, and *R. pallescens*. Furthermore, additional research is needed to address this question.

Insects have an immune system that gives them protection from pathogens and keeps their systems in balance. While insects have a natural resistance to *T. cruzi* (Mwangi et al., 2023; Abreu et al., 2022), this also has strategies to modulate the immune response of the insect in its favor, such as the production of nitrite/nitrate, allowing it to establish itself (Fredensborg et al., 2020). The outcome of the microbiota of the insects infected with *T. cruzi* indicates a variation in distribution and abundance between insects that have *T. cruzi* as part of their microbiota and those that do not. This may occur because native bacteria compete for space and resources. Orantes et al. (2018) mention that, in the midgut, an anaerobic environment, bacteria can regulate extracellular glucose levels to facilitate or prevent the colonization of competitors such as *T. cruzi* and by the interaction between insects and microbiota-parasites (Soares et al., 2015). Further research is needed to understand if there are differences in microbiome behavior based on *T. cruzi* lineages. It is important to note that only discrete typing unit I (DTU TcI) isolates were collected from domestic, peridomestic, and sylvatic hosts and vectors in and around several neighboring communities in Loja Province, Ecuador (Ocana-Mayorga et al., 2010; Costales et al., 2015).

This study provides a general overview of the bacterial intestinal microbiota of *R. ecuadoriensis* without examining other microbial kingdoms. Guarneri Alessandra and Schaub (2021) report the presence of various fungi in the intestine of triatomines, so the role that fungi play as symbionts of *R. ecuadoriensis* could be considered for future research (Gurung et al., 2019). Similarly, other biological entities like the *Triatoma* virus, the only virus reported to affect triatomines to date (Montoya-Porras et al., 2018; Mwangi et al., 2023), could be explored through the tests carried out by Abreu et al. (2022). *R. ecuadoriensis* indicates that it does not affect the *Triatoma* virus. Additional research focused on this biological entity could lead to its modification and use for selective control of more species of triatomines.

The diversity indices used in this study, including Shannon and Simpson indices and species richness, reveal low, moderate, and high diversity of genera and species. Extrapolation lines on the rarefaction curve suggest that additional genera may be discovered with more sequencing reads, revealing significant differences in genus diversity between wild and laboratory-raised samples, as well as among communities and genera. The microbiota of *R. ecuadoriensis* plays a crucial role in its biology and the transmission of Chagas disease. Understanding its interactions with the host and the relationship between the intestinal microbiota and *T. cruzi* is essential. While progress has been made, a complete understanding of the complex interactions among the various microbial kingdoms in the triatomine intestinal microbiota is still needed. This study, however, represents a vital first step in uncovering these interactions and advancing new methodologies.

### 4.1 Limitations

Given the sample pool methodology, individual information on the insects is lost; in the same way, bacteria that present a very low abundance are underestimated, and there is still no information on the insects from the other provinces of Ecuador and Peru.

## Data Availability

The raw data supporting the conclusions of this article will be made available by the authors, without undue reservation.
